# Supplementary material for: SEM-2/SoxC regulates multiple aspects of C. elegans postembryonic mesoderm development
Source: PLoS Genet. 2025 Jan 21;21(1):e1011361. doi: 10.1371/journal.pgen.1011361 (PMC11785321; doi:10.1371/journal.pgen.1011361)
Supplement: S1 Table — (DOCX) [file pgen.1011361.s001.docx]

**Supplementary table 1. *C. elegans* strains used in this study**

| **Strain ID** | **Genotype** |  |
| --- | --- | --- |
| **Strains carrying the SEM-2 P158S mutation** | | |
| LW5780 | *sem-2(jj152) I* | |
| LW5914 | *sem-2(jj320) I* | |
| LW5915 | *sem-2(jj321) I* | |
| **Strains carrying other *sem-2* mutations** | | |
| LW6501 | *hT2[qIs48] (I;III)/sem-2(ok2422) I; jjIs3900* [*hlh-8p::nls::mCherry::lacZ+myo-2::mCherry] IV* | |
| LW1221 | *sem-2(n1343) I* | |
| LW2734 | *sem-2(n1343) I; ccIs4438(intrinsic CC:::gfp) III; ayIs2(egl-15p::gfp) IV; sma-9(cc604) ayIs6(hlh-8p::gfp) X* | |
| LW3880 | *sem-2(n1343) I; ccIs4438(intrinsic CC:::gfp) III; ayIs2(egl-15p::gfp) IV; ayIs6(hlh-8p::gfp) X* | |
| **Strains carrying endogenously-tagged SEM-2** | | |
| LW6287 | *sem-2(jj382[gfp::2xflag::sem-2]) I* | |
| LW6282 | *sem-2(jj382[gfp::2xflag::sem-2]) I; jjIs3900* [*hlh-8p::nls::mCherry::lacZ+myo-2::mCherry] IV* | |
| LW6466 | *sem-2(jj382[gfp::2xflag::sem-2]) I; jjIs3900* [*hlh-8p::nls::mCherry::lacZ+myo-2::mCherry] IV; sma-9(cc604) X* | |
| LW6467 | *sem-2(jj382 jj417[gfp::2xflag::sem-2 P158S] I* | |
| LW6537 | *sem-2(jj382 jj417[gfp::2xflag::sem-2 P158S] I; jjIs3900* [*hlh-8p::nls::mCherry::lacZ+myo-2::mCherry] IV* | |
| **Strains carrying various reporters** | | |
| LW3949 | *jjIs3900* [*hlh-8p::nls::mCherry::lacZ+myo-2::mCherry] IV* | |
| LW0081 | *ccIs4438(intrinsic CC:::gfp) III; ayIs2(egl-15p::gfp) IV; ayIs6(hlh-8p::gfp) X* | |
| SYS668 | *let-381(dev205[mNeonGreen::let-381]) I* | |
| IX4506 | *mls-2(vy248[mNeonGreen::mls-2]) X* | |
| **Strains used for examining the Susm phenotype of the SEM-2 P158S mutation** | | |
| LW2598 | *arIs37[secreted CC::gfp] I; cup-5(ar465) III; sma-9(cc604) X* | |
| LW5924 | *sem-2(jj152) arIs37[secreted CC::gfp] I; cup-5(ar465) III; sma-9(cc604) X* | |
| LW5903 | *sem-2(jj152) arIs37[secreted CC::gfp] I; cup-5(ar465) III; sma-9(cc604) X; jjIs1647[N-GFP::sem-2 fosmid + pRF4]* | |
| **Strains for examining the M lineage phenotypes in SEM-2 P158S mutants** | | |
| LW6928 | *let-381(dev205[mNeonGreen::let-381]) I; jjIs3900 [hlh-8p::nls::mCherry::lacZ+myo-2::mCherry] IV* | |
| LW6727 | *sem-2(jj476[SEM-2 P158S]) let-381(dev205[mNeonGreen::let-381] I; jjIs3900 [hlh-8p::nls::mCherry::lacZ+myo-2::mCherry] IV* | |
| LW6929 | *let-381(dev205[mNeonGreen::let-381]) I; jjIs3900 [hlh-8p::nls::mCherry::lacZ+myo-2::mCherry] IV; sma-9(cc604) X* | |
| LW6934 | *sem-2(jj476[SEM-2 P158S]) let-381(dev205[mNeonGreen::let-381]) I; jjIs3900 [hlh-8p::nls::mCherry::lacZ+myo-2::mCherry] IV; sma-9(cc604) X isolate #1* | |
| LW6935 | *sem-2(jj476[SEM-2 P158S]) let-381(dev205[mNeonGreen::let-381]) I; jjIs3900 [hlh-8p::nls::mCherry::lacZ+myo-2::mCherry] IV; sma-9(cc604) X isolate #2* | |
| LW5847 | *sem-2(jj152) I; ccIs4438[intrinsic CC::gfp] III isolate #1* | |
| LW5848 | *sem-2(jj152) I; ccIs4438[intrinsic CC::gfp] III isolate #2* | |
| LW5845 | *sem-2(jj152) I; ccIs4438[intrinsic CC::gfp] III; arIs2[egl-15p::gfp] IV isolate #1* | |
| LW5846 | *sem-2(jj152) I; ccIs4438[intrinsic CC::gfp] III; arIs2[egl-15p::gfp] IV isolate #2* | |
| LW5931 | *sem-2(jj152) I; ayIs2(egl-15p::gfp) IV; ayIs6(hlh-8p::gfp) X isolate #1* | |
| LW5932 | *sem-2(jj152) I; ayIs2(egl-15p::gfp) IV; ayIs6(hlh-8p::gfp) X isolate #2* | |
| LW6474 | *sem-2(jj321) I; jjIs3900 [hlh-8p::nls::mCherry::lacZ+myo-2::mCherry] IV isolate #1* | |
| LW6475 | *sem-2(jj321) I; jjIs3900 [hlh-8p::nls::mCherry::lacZ+myo-2::mCherry] IV isolate #2* | |
| LW4072 | *jjIs3644[egl-15p::rfp(pJKL737)+dpy-20(+)] II; hlh-29p::gfp [TLM908] III* | |
| LW5996 | *sem-2(jj321) I; jjIs3644[egl-15p::rfp(pJKL737)+dpy-20(+)] II; hlh-29p::gfp [TLM908] III isolate #1* | |
| LW5997 | *sem-2(jj321) I; jjIs3644[egl-15p::rfp(pJKL737)+dpy-20(+)] II; hlh-29p::gfp [TLM908] III isolate #2* | |
| LW5998 | *sem-2(jj321) I; jjIs3644[egl-15p::rfp(pJKL737)+dpy-20(+)] II; hlh-29p::gfp [TLM908] III isolate #3* | |
| LW3189 | *vsIs4[rgs-2p::gfp]* | |
| LW6473 | *sem-2(jj321) I; vsIs4[rgs-2p::gfp]* | |
| LW3430 | *ccIs4443[arg-1p::gfp] IV* | |
| LW6385 | *sem-2(jj321) I; ccIs4443[arg-1p::gfp] IV isolate #1* | |
| LW6386 | *sem-2(jj321) I; ccIs4443[arg-1p::gfp] IV isolate #2* | |
| LW1379 | *jjIs1379[pJKL743(NdEbox::nls::gfp+unc-119(+)]; unc-119(ed4) III* | |
| LW6415 | *sem-2(jj321) I; jjIs1379[pJKL743(NdEbox::nls::gfp+unc-119(+)]* | |
| LW6683 | *jjIs3900 [hlh-8p::nls::mCherry::lacZ+myo-2::mCherry] IV; mls-2(vy248[mNeonGreen::mls-2]) X* | |
| LW6684 | *sem-2(jj321) I; jjIs3900 [hlh-8p::nls::mCherry::lacZ+myo-2::mCherry] IV; mls-2(vy248[mNeonGreen::mls-2]) X isolate #1* | |
| LW6685 | *sem-2(jj321) I; jjIs3900 [hlh-8p::nls::mCherry::lacZ+myo-2::mCherry] IV; mls-2(vy248[mNeonGreen::mls-2]) X isolate #2* | |
| **Strains with transgenic *hlh-8* promoter deletions** | | |
| LW0649 | *jjEx649[pAYL11(hlh-8p(517bp)::gfp) + PRF4] line #1* |  |
| LW0652 | *jjEx652[pAYL11(hlh-8p(517bp)::gfp) + PRF4] line #2* |  |
| LW0793 | *jjEx793[pAYL11(hlh-8p(517bp)::gfp) + PRF4] line #3* |  |
| LW0787 | *jjEx787[pAYL21(hlh-8p(deletion)::gfp + PRF4] line #1* |  |
| LW0790 | *jjEx790[pAYL21(hlh-8p(deletion)::gfp + PRF4] line #2* |  |
| LW0366 | *jjEx366[pAYL21(hlh-8p(deletion)::gfp + PRF4] line #3* |  |
| LW0798 | *jjEx798[pAYL22(hlh-8p(deletion)::gfp + PRF4] line #1* |  |
| LW0802 | *jjEx802[pAYL22(hlh-8p(deletion)::gfp + PRF4] line #2* |  |
| LW0651 | *jjEx651[pAYL23(hlh-8p(deletion)::gfp + PRF4] line #1* |  |
| LW0788 | *jjEx788[pAYL23(hlh-8p(deletion)::gfp + PRF4] line #2* |  |
| LW0801 | *jjEx801[pAYL23(hlh-8p(deletion)::gfp + PRF4] line #3* |  |
| LW0796 | *jjEx796[pAYL24(hlh-8p(deletion)::gfp + PRF4] line #1* |  |
| LW0803 | *jjEx803[pAYL24(hlh-8p(deletion)::gfp + PRF4] line #2* |  |
| LW0808 | *jjEx808[pAYL24(hlh-8p(deletion)::gfp + PRF4] line #3* |  |
| LW0811 | *jjEx811[pAYL25(hlh-8p(deletion)::gfp + PRF4] line #1* |  |
| LW0800 | *jjEx800[pAYL31(hlh-8p(deletion)::gfp + PRF4] line #1* |  |
| LW0806 | *jjEx806[pAYL31(hlh-8p(deletion)::gfp + PRF4] line #2* |  |
| LW0785 | *jjEx785[pAYL32(hlh-8p(deletion)::gfp + PRF4] line #1* |  |
| LW0791 | *jjEx791[pAYL32(hlh-8p(deletion)::gfp + PRF4] line #2* |  |
| LW0792 | *jjEx792[pAYL32(hlh-8p(deletion)::gfp + PRF4] line #3* |  |
| LW0795 | *jjEx795[pAYL32(hlh-8p(deletion)::gfp + PRF4] line #4* |  |
| LW0789 | *jjEx789[pAYL33(hlh-8p(deletion)::gfp + PRF4] line #1* |  |
| LW0794 | *jjEx794[pAYL33(hlh-8p(deletion)::gfp + PRF4] line #2* |  |
| LW0786 | *jjEx786[pAYL35(hlh-8p(deletion)::gfp + PRF4] line #1* |  |
| LW0804 | *jjEx804[pAYL35(hlh-8p(deletion)::gfp + PRF4] line #2* |  |
| LW0805 | *jjEx805[pAYL35(hlh-8p(deletion)::gfp + PRF4] line #3* |  |
| LW6717 | *jjEx6717[pAYL11(hlh-8p(517bp)::gfp) + pJKL449(myo-2p::gfp)]; jjIs3900* [*hlh-8p::nls::mCherry::lacZ + myo-2::mCherry] IV* |  |
| LW6720 | *jjEx6720[pAYL35(hlh-8p(deletion)::gfp) + pJKL449(myo-2p::gfp)]; jjIs3900* [*hlh-8p::nls::mCherry::lacZ + myo-2::mCherry] IV line #1* |  |
| LW6751 | *Ex[pAYL35(hlh-8p(deletion)::gfp) + pJKL449(myo-2p::gfp)]; jjIs3900* [*hlh-8p::nls::mCherry::lacZ + myo-2::mCherry] IV line #2* |  |
| LW6718 | *jjEx6718[pAYL32(hlh-8p(deletion)::gfp) + pJKL449(myo-2p::gfp)]; jjIs3900* [*hlh-8p::nls::mCherry::lacZ + myo-2::mCherry] IV line #1* |  |
| LW6719 | *jjEx6719[pAYL32(hlh-8p(deletion)::gfp) + pJKL449(myo-2p::gfp)]; jjIs3900* [*hlh-8p::nls::mCherry::lacZ + myo-2::mCherry] IV line #2* |  |
| **Strains with the endogenous *hlh-8* transcriptional reporter** | | |
| LW6459 | *hlh-8(jj422[hlh-8p::hlh-8::sl2::nls::gfp::nls::hlh-8 3’ UTR]) X* |  |
| LW6476 | *jjIs3900* [*hlh-8p::nls::mCherry::lacZ+myo-2::mCherry] IV; hlh-8(jj422[hlh-8p::hlh-8::sl2::nls::gfp::nls::hlh-8 3’ UTR]) X isolate #1* |  |
| LW6477 | *jjIs3900* [*hlh-8p::nls::mCherry::lacZ+myo-2::mCherry] IV; hlh-8(jj422[hlh-8p::hlh-8::sl2::nls::gfp::nls::hlh-8 3’ UTR]) X isolate #2* |  |
| LW6478 | *sem-2(jj321) I; jjIs3900* [*hlh-8p::nls::mCherry::lacZ+myo-2::mCherry] IV; hlh-8(j422[hlh-8p::hlh-8::sl2::nls::gfp::nls::hlh-8 3’ UTR]) X isolate #1* |  |
| LW6479 | *sem-2(jj321) I; jjIs3900* [*hlh-8p::nls::mCherry::lacZ+myo-2::mCherry] IV; hlh-8(j422[hlh-8p::hlh-8::sl2::nls::gfp::nls::hlh-8 3’ UTR]) X isolate #2* |  |
| **Strains with endogenous *hlh-8* promoter mutations** | | |
| LW6498 | *hlh-8(jj445 jj422[hlh-8p(13bp mutation at -272bp to -259bp in the hlh-8 promoter)::hlh-8::sl2::nls::gfp::nls::hlh-8 3’ UTR]) X* |  |
| LW6499 | *hlh-8(jj446 jj422[hlh-8p(13bp mutation at -272bp to -259bp in the hlh-8 promoter)::hlh-8::sl2::nls::gfp::nls::hlh-8 3’ UTR] X* |  |
| LW6686 | *hlh-8(jj483 jj422[hlh-8p(10bp mutation at -221bp to -211bp and 13bp mutation at -272bp to -259bp in the hlh-8 promoter)::hlh-8::sl2::nls::gfp::nls::hlh-8 3’ UTR] X* |  |
| LW6528 | *jjIs3900 [hlh-8p::nls::mCherry::lacZ + myo-2::mCherry] IV; hlh-8(jj445 jj422[hlh-8p(13bp mutation at -272bp to -259bp in the hlh-8 promoter)::hlh-8::sl2::nls::gfp::nls::hlh-8 3’ UTR]) X isolate #1* |  |
| LW6529 | *jjIs3900* [*hlh-8p::nls::mCherry::lacZ + myo-2::mCherry] IV; hlh-8(jj445 jj422[hlh-8p13bp mutation at at -272bp to -259bp in the hlh-8 promoter)::hlh-8::sl2::nls::gfp::nls::hlh-8 3’ UTR]) X isolate #2* |  |
| LW6822 | *jjIs3900* [*hlh-8p::nls::mCherry::lacZ + myo-2::mCherry] IV; hlh-8(jj446 jj422[hlh-8p(13bp mutation at at -272bp to -259bp in the hlh-8 promoter)::hlh-8::sl2::nls::gfp::nls::hlh-8 3’ UTR] X isolate #1* |  |
| LW6699 | *jjIs3900* [*hlh-8p::nls::mCherry::lacZ + myo-2::mCherry] IV; hlh-8(jj483 jj422[hlh-8p(10bp mutation at at -221bp to -211bp and 13bp mutation at -272bp to -259bp in the hlh-8 promoter)::hlh-8::sl2::nls::gfp::nls::hlh-8 3’ UTR] X isolate #1* |  |
| LW6700 | *jjIs3900* [*hlh-8p::nls::mCherry::lacZ + myo-2::mCherry] IV; hlh-8(jj483 jj422[hlh-8p(10bp mutation at -221bp to -211bp and 13bp mutation at -272bp to -259bp in the hlh-8 promoter)::hlh-8::sl2::nls::gfp::nls::hlh-8 3’ UTR] X isolate #2* |  |
