# Supplementary material for: SEM-2/SoxC regulates multiple aspects of C. elegans postembryonic mesoderm development
Source: PLoS Genet. 2025 Jan 21;21(1):e1011361. doi: 10.1371/journal.pgen.1011361 (PMC11785321; doi:10.1371/journal.pgen.1011361)
Supplement: S2 Table — (DOCX) [file pgen.1011361.s002.docx]

**­Supplementary table 2. Plasmids generated in this study.**

| **Plasmid ID** | **Details** |
| --- | --- |
| ***hlh-8* promoter deletion constructs** | |
| pAYL11 | *517bp(-517* to -1*) hlh-8p::gfp::unc-54 3’ UTR* |
| pAYL21 | *517bp(deletion of -401 to -350) hlh-8p::gfp::unc-54 3’ UTR* |
| pAYL22 | *517bp(deletion of -351 to -300) hlh-8p::gfp::unc-54 3’ UTR* |
| pAYL23 | *517bp(deletion of -301 to -250) hlh-8p::gfp::unc-54 3’ UTR* |
| pAYL24 | *517bp(deletion of -251 to -200) hlh-8p::gfp::unc-54 3’ UTR* |
| pAYL25 | *517bp(deletion of -201 to -150) hlh-8p::gfp::unc-54 3’ UTR* |
| pAYL31 | *517bp(deletion of -300 to -281) hlh-8p::gfp::unc-54 3’ UTR* |
| pAYL32 | *517bp(deletion of -280 to -261) hlh-8p::gfp::unc-54 3’ UTR* |
| pAYL33 | *517bp(deletion of -260 to -241) hlh-8p::gfp::unc-54 3’ UTR* |
| pAYL34 | *517bp(deletion of -240 to -221) hlh-8p::gfp::unc-54 3’ UTR* |
| pAYL35 | *517bp(deletion of -220 to -201) hlh-8p::gfp::unc-54 3’ UTR* |
| **Plasmids used for CRISPR** | |
| pMDB15 | sgRNA plasmid #1 for making the SEM-2 P158S mutation [*sem-2(jj321), sem-2(jj320), and sem-2(jj417)] (in pRB1017)*  MDB-19: TCTTGATTCTTCTTTGGCTTCTTGCG  MDB-20: AAACCGCAAGAAGCCAAAGAAGAATC |
| pVG1 | sgRNA plasmid #2 for making the SEM-2 P158S mutation [*sem-2(jj321), sem-2(jj320), and sem-2(jj417)] (in pRB1017)*  JKL-1912: TCTTGCGTGGCTTGTATTTGTAGTC  JKL-1913: AAACGACTACAAATACAAGCCACGC |
| pMDB27 | sgRNA plasmid #1 for generating *gfp::2xflag::sem-2 [sem-2(jj382)] (in pRB1017)*  MDB-32: TCTTGAATCGGCGGCATCATATGC  MDB-33: AAACGCATATGATGCCGCCGATTC |
| pMDB29 | sgRNA plasmid #2 for generating *gfp::2xflag::sem-2 [sem-2(jj382)] (in pRB1017)*  MDB-44: TCTTGCCAACTTCATGCTGGATTG  MDB-45: AAACCAATCCAGCATGAAGTTGGC |
| pMDB28 | *Repair template for generating gfp::2xflag::sem-2 [sem-2(jj382)]* |
| pMDB33 | sgRNA plasmid #1 for generating the endogenous *hlh-8* transcriptional reporter *hlh-8(jj422[hlh-8p::hlh-8::sl2::nls::gfp::nls::hlh-8 3’UTR])* (in *pRB1017*)  MDB-69: TCTTGGAATTGGGATTTGGAGTTGAGA  MDB-70: AAACTCTCAACTCCAAATCCCAATTCC |
| pMDB34 | sgRNA plasmid #2 for generating the endogenous *hlh-8* transcriptional reporter *hlh-8(jj422[hlh-8p::hlh-8::sl2::nls::gfp::nls::hlh-8 3’UTR])* (in *pRB1017*)  MDB-71: TCTTGTGGAGTGAGTTGATTTGGATT  MDB-72: AAACAATCCAAATCAACTCACTCCAC |
| pMDB35 | sgRNA plasmid #3 for generating the endogenous *hlh-8* transcriptional reporter *hlh-8(jj422[hlh-8p::hlh-8::sl2::nls::gfp::nls::hlh-8 3’UTR])* (in *pRB1017*)  MDB-73: TCTTGTCCAAATCAACTCACTCCAATTCAC  MDB-74: AAACGTGAATTGGAGTGAGTTGATTTGGAC |
| pMDB36 | sgRNA plasmid #4 for generating the endogenous *hlh-8* transcriptional reporter *hlh-8(jj422[hlh-8p::hlh-8::sl2::nls::gfp::nls::hlh-8 3’UTR])* (in *pRB1017*)  MDB-75: TCTTGTTATTGCCAGTGAATTGGAG  MDB-76: AAACCTCCAATTCACTGGCAATAAC |
| pMDB37 | Repair template for generating *hlh-8(jj422[hlh-8p::hlh-8::sl2::nls::gfp::nls::hlh-8 3’UTR])* |

All plasmids were verified by Sanger sequencing.
