# Supplementary material for: SEM-2/SoxC regulates multiple aspects of C. elegans postembryonic mesoderm development
Source: PLoS Genet. 2025 Jan 21;21(1):e1011361. doi: 10.1371/journal.pgen.1011361 (PMC11785321; doi:10.1371/journal.pgen.1011361)
Supplement: S3 Table — (DOCX) [file pgen.1011361.s003.docx]

**Supplementary table 3. Oligonucleotides used in this study**

| **Oligo ID** | **Sequence** |  |
| --- | --- | --- |
| **Repair oligo for generating SEM-2 P158S** | | |
| MDB-21 | | For generating *sem-2(jj320)*, *sem-2(jj321), sem-2(jj417) and sem-2(jj476)*  AGGAAATACTGTAACCGGATTTTTCGAATAATTGTATTGTAATTTTAAATTTTTTCAGGAATACTCAGATTACAAGTACAAGCCACGTAAAAAGCCGAAAAAGAACCCAGATGGAACACTTCAGCAGCCAGCTCAACCCCAAGCTC |
| **Repair oligos for generating mutations in the *hlh-8* promoter** | |  |
| MDB-97 | For generating a 13bp mutation *at* -272bp to -259bp in the *hlh-8* promoter [*hlh-8(jj445) and hlh-8(jj446)]*  TTCTACCTTCACTCTCAAATTCTTTTTTCAGCGGTAATTTTTCAACTATCGAGGCGCCGCAGGCCTCATGAATAACTGTCAGCATAGAGTTCTCACCCGTCCCCTTTCTTTTCACCGAG |  |
| MDB-146 | For generating a 10bp mutation at -221bp to -211bp and a 13bp mutation *at* -272bp to -259bp in the *hlh-8* promoter [*hlh-8(jj483)]*  GTGTTAGTGTAGGTTGCTTTGCTTCTACCTTCACTCTCAAATTCTTTTTTCAGCGGTAATTTTTCAACTATCGAGGCGCCGCAGGCCTCATGAATAACTGTCAGCATAGAGTTCTCACCCGTCCCCCGAGGCCGGACCGAGCAGTCACGAGAGAAAGAAGAAGAAGCGGACCGCTGCAGAGATTCTTCGTAGCGG |  |
| **sgRNA for CRISPR** | |  |
| sgRNA-MDB-1 | guide #1 targeting the *hlh-8* promoter to generate *hlh-8(jj445), hlh-8(jj446),* and *hlh-8(jj483)*  CGATAGTTGAAAAATTACCG |  |
| sgRNA-MDB-2 | guide #2 targeting the *hlh-8* promoter to generate *hlh-8(jj445), hlh-8(jj446),* and *hlh-8(jj483)*  GGACGGGTGAGAACTCTATG |  |
| sgRNA-MDB-7 | guide #1 targeting *sem-2* to generate *sem-2(jj476)*  TTCTTCTTTGGCTTCTTGCG | |
| sgRNA-MDB-8 | guide #2 targeting *sem-2* to generate *sem-2(jj476)*  CGTGGCTTGTATTTGTAGTC | |
| **For genotyping *sem-2(jj152)* and *sem-2(jj320/1)* i.e. SEM-2 P158S** | |  |
| JKL-1910 | GTCAGTGTAGGAGGTAGGTG |  |
| JKL-1911 | GGCATCTTTTGTGCATGACTC |  |
| **For amplification to genotype for *sem-2(jj152)* and *sem-2(jj320/1)* i.e. SEM-2 P158S** | | |
| JKL-1910 | | GTCAGTGTAGGAGGTAGGTG |
| JKL-1911 | | GGCATCTTTTGTGCATGACTC |
| **For genotyping *sem-2(ok2422)*** | | |
| CXT-46 | | CAGATATCAAATGGATCTCC |
| CXT-47 | | GTCCTTTTGACAGCTTATCAC |
| CXT-48 | | CAATGCATCGCTCCATGGATAA |
| **For genotyping *sem-2(jj382)*** | | |
| MDB-P15 (for screening and homozygosing) | | ACGAGGATTGGGACAACTCC |
| JKL-1922 (for screening) | | CTCGAGAGAGAGAGAGAGAAATG |
| MDB-65 (for homozygosing) | | CTGCCGAAATTCGGTCTCCTG |
| CXT-47 (for homozygosing) | | GTCCTTTTGACAGCTTATCAC |
| **For genotyping *hlh-8(jj380/1)*** | |  |
| MDB-56 | GAAATCATGCTGAACTATGG |  |
| MDB-57 | TTCGAACCACGCGTTCTCC |  |
| MDB-58 | ATTCATGACTTCTTCTAAGC |  |
| **For genotyping *hlh-8(jj445/6)*** | |  |
| MDB-56 | GAAATCATGCTGAACTATGG |  |
| MDB-57 | TTCGAACCACGCGTTCTCC |  |
| MDB-99 | TTTCAACTATCGAGGCGCC |  |
| **For genotyping *hlh-8(jj483)*** | |  |
| MDB-56 | GAAATCATGCTGAACTATGG |  |
| MDB-57 | TTCGAACCACGCGTTCTCC |  |
| MDB-145 | GACTGCTCGGTCCGGCCTCGG |  |
| **For genotyping *hlh-8(jj422)*** | |  |
| MDB-89  (for screening) | GTCACTAGTCTTGGATGTCAGTACC |  |
| MDB-90  (for screening) | GAAGAAGCGTAAGGTACCGG |  |
| MDB-84 (for homozygosing) | AGAAGAATTTCATGAACTGTGTCCC |  |
| MDB-85 (for homozygosing) | CAATGGGCTTACTAATAGTCTCTTGC |  |
| MDB-86 (for homozygosing) | GGAGAACTTGTGTCCGTTGAC |  |
