# Supplementary figures and images for: SEM-2/SoxC regulates multiple aspects of C. elegans postembryonic mesoderm development
Source: PLoS Genet. 2025 Jan 21;21(1):e1011361. doi: 10.1371/journal.pgen.1011361 (PMC11785321; doi:10.1371/journal.pgen.1011361)

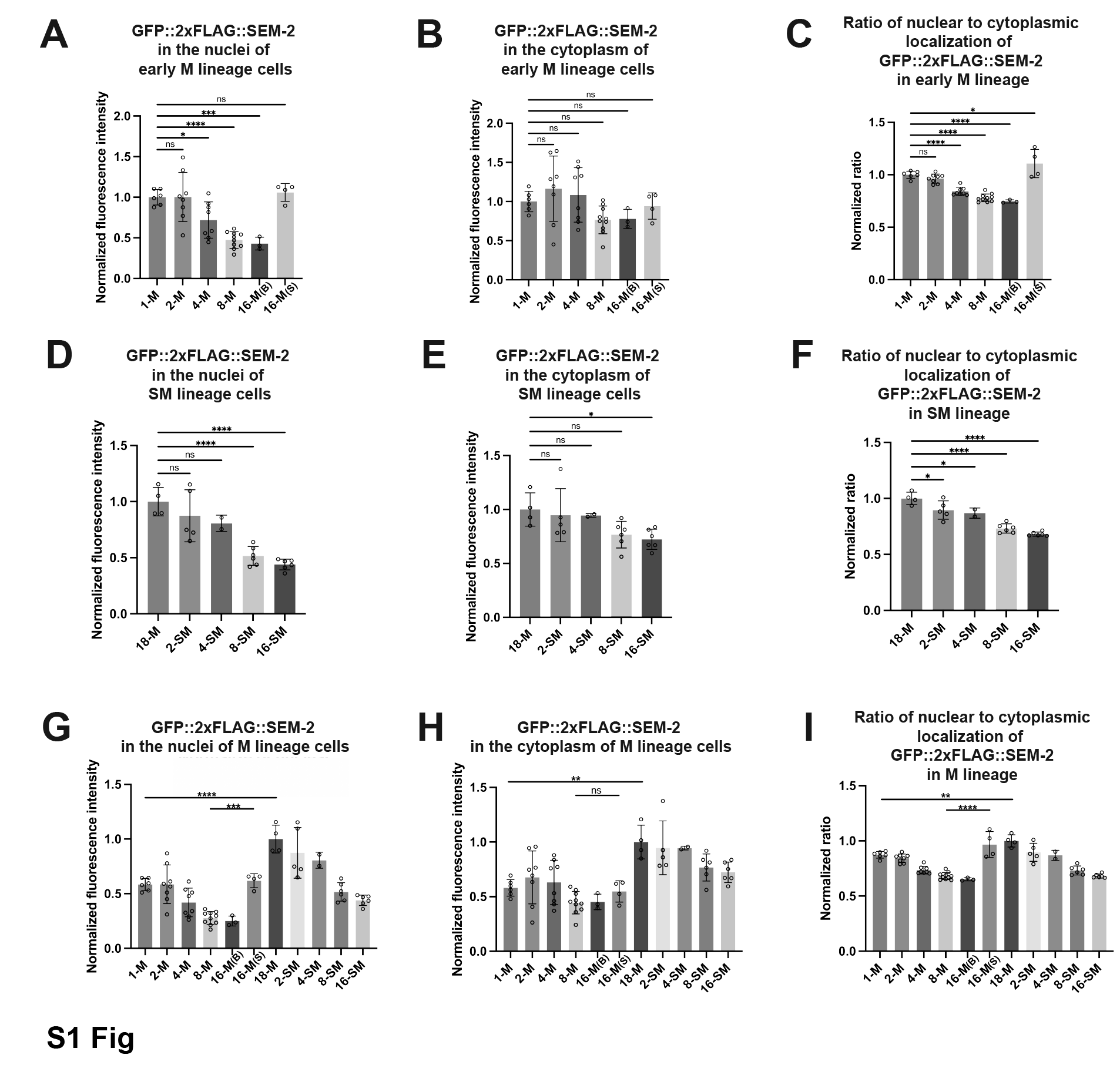

Supplement: S1 Fig — A–C) Quantification of GFP::2xFLAG::SEM-2 fluorescence intensity in the nuclei (A, D, G), the cytoplasm (B, E, H), and the ratio of nuclear signal to cytoplasmic signal (C, F, I) in the early M lineage (A, B, C), the SM lineage (D, E, F), and throughout M lineage development (G, H, I). All images were taken at the same exposure and same magnification. Each dot represents a cell scored. Data for GFP::2xFLAG::SEM-2 expression at the 16-M stage in M-derived BWMs and SM mother cells are denoted 16-M(B) and 16-M(S), respectively. For graphs D-I, only data for the SMs at the 18-M stage are shown. For panels C, F, and I, the ratios were calculated by dividing the nuclear GFP intensity by the cytoplasmic GFP intensity. For panels A–C, data were normalized to the 1-M stage. For panels D–I, data were normalized to the 18-M stage. Statistical analysis was done using one-way ANOVA with Dunnett’s test. **** P<0.0001, *** P<0.001, ** P<0.01, * P<0.05, ns, not significant. All the corresponding data shown in panels A-C and D-F were combined and shown in panels G-I. To prevent the graph from being too crowded, only P values not shown in panels A-F are shown in G-I. There is a gradual decrease in the level of nuclear GFP::2xFLAG::SEM-2 in the early M lineage after the 2-M stage (A). At the 16-M stage, there is an upregulation of nuclear GFP::2xFLAG::SEM-2 in the SM mothers (A). At the 18-M stage, this increase in nuclear GFP::2xFLAG::SEM-2 persists in the SMs but becomes undetectable in the BWMs (including in the SM sister cells, which transiently express GFP::2xFLAG::SEM-2) upon terminal differentiation. There is then a gradual decrease in the level of nuclear GFP::2xFLAG::SEM-2 signal in the SM descendants (D). The level of cytoplasmic GFP::2xFLAG::SEM-2 signal appears relatively stable within the early M lineage (B), and within the SM lineage (E). The ratio of nuclear to cytoplasmic localization of GFP::2xFLAG::SEM-2 in the M lineage follows the pattern of change of nuclear GFP:: [file pgen.1011361.s004.tif]

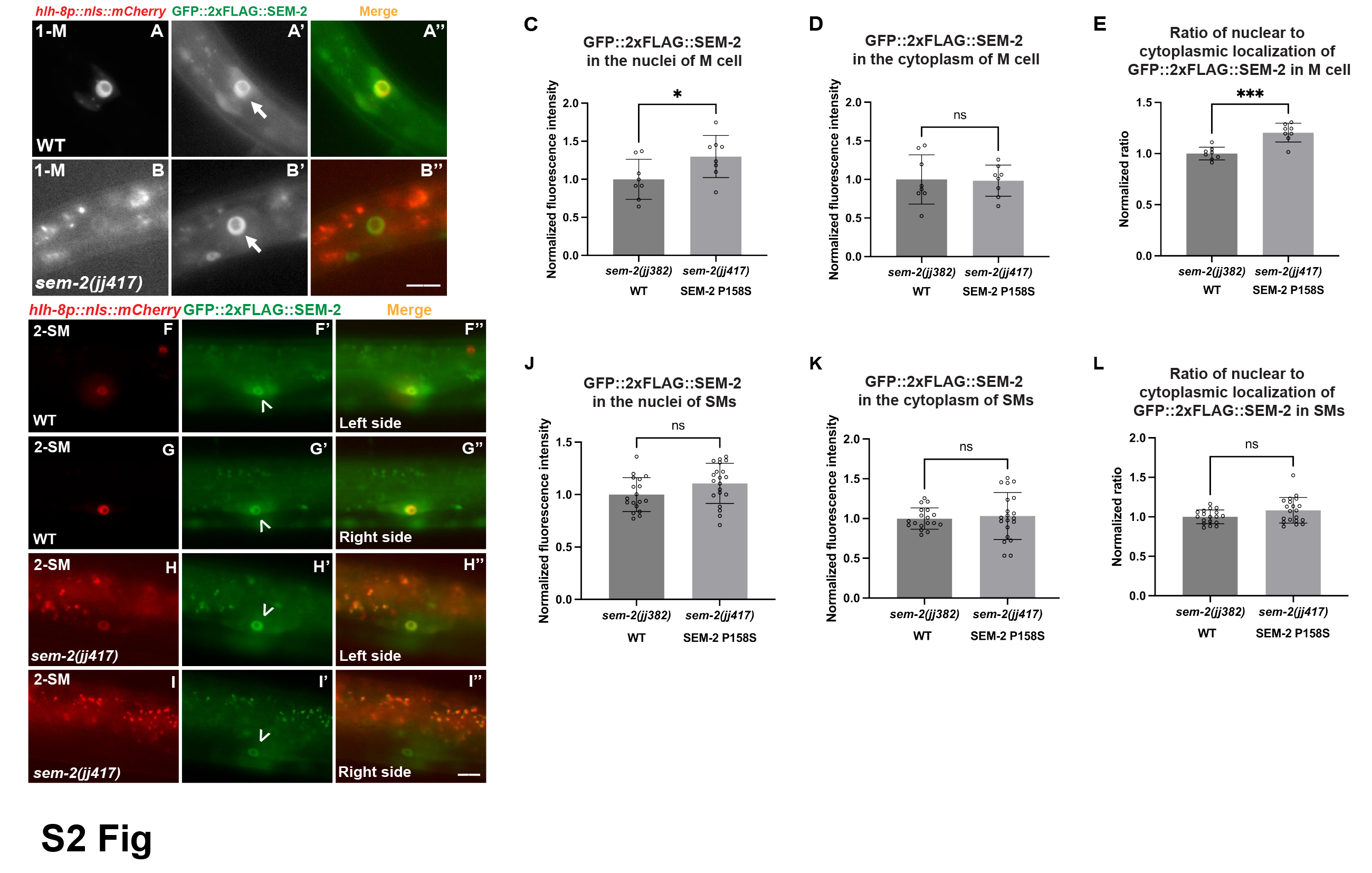

Supplement: S2 Fig — A–B”) Fluorescence images showing GFP::2xFLAG::SEM-2 (A’–B’) in the M mesoblast cell labelled by the hlh-8p::nls::mCherry reporter (A–B) at the 1-M stage in WT (A–A”) and sem-2(jj417[SEM-2 P158S]) (B–B”) hermaphrodites. (A”–B”) are the corresponding merged images. The GFP::2xFLAG::SEM-2 images were taken at the same exposure and same magnification. C–E) Quantification of GFP::2xFLAG::SEM-2 in the nuclei (C), the cytoplasm (D), and the ratio of nuclear to cytoplasmic signal (E) in the M mesoblast cell of wild-type and sem-2(jj417[SEM-2 P158S]) animals. F–I”) Fluorescence images of a wild-type (sem-2(jj382)) animal (F–G”) and a sem-2(jj417[P158S]) mutant animal (H–I”), showing GFP::2xFLAG::SEM-2 (F’–I’), hlh-8p::nls::mCherry (F–I), and the corresponding merged images (F”–I”) in the two SMs. All GFP::2xFLAG::SEM-2 images were taken at the same exposure and same magnification, while hlh-8p::nls::mCherry images in H and I were taken using a longer exposure than those in F and J. J–L) Quantification of GFP::2xFLAG::SEM-2 in the nuclei (J), the cytoplasm (K), and the ratio of nuclear to cytoplasmic signal (L) in the SMs of wild-type and sem-2(jj417[SEM-2 P158S]) animals. For panels E and L, the ratios were calculated by dividing the nuclear GFP intensity by the cytoplasmic GFP intensity. Each dot represents a cell scored. Data are normalized to WT. Statistical significance was calculated by performing unpaired two-tailed Student’s t-tests. *** P<0.001, * P<0.05, ns, not significant. Scale bars represent 10 μm. Arrows point to the M mesoblast cell, while arrowheads point to the SM cell. (TIF) [file pgen.1011361.s005.tif]

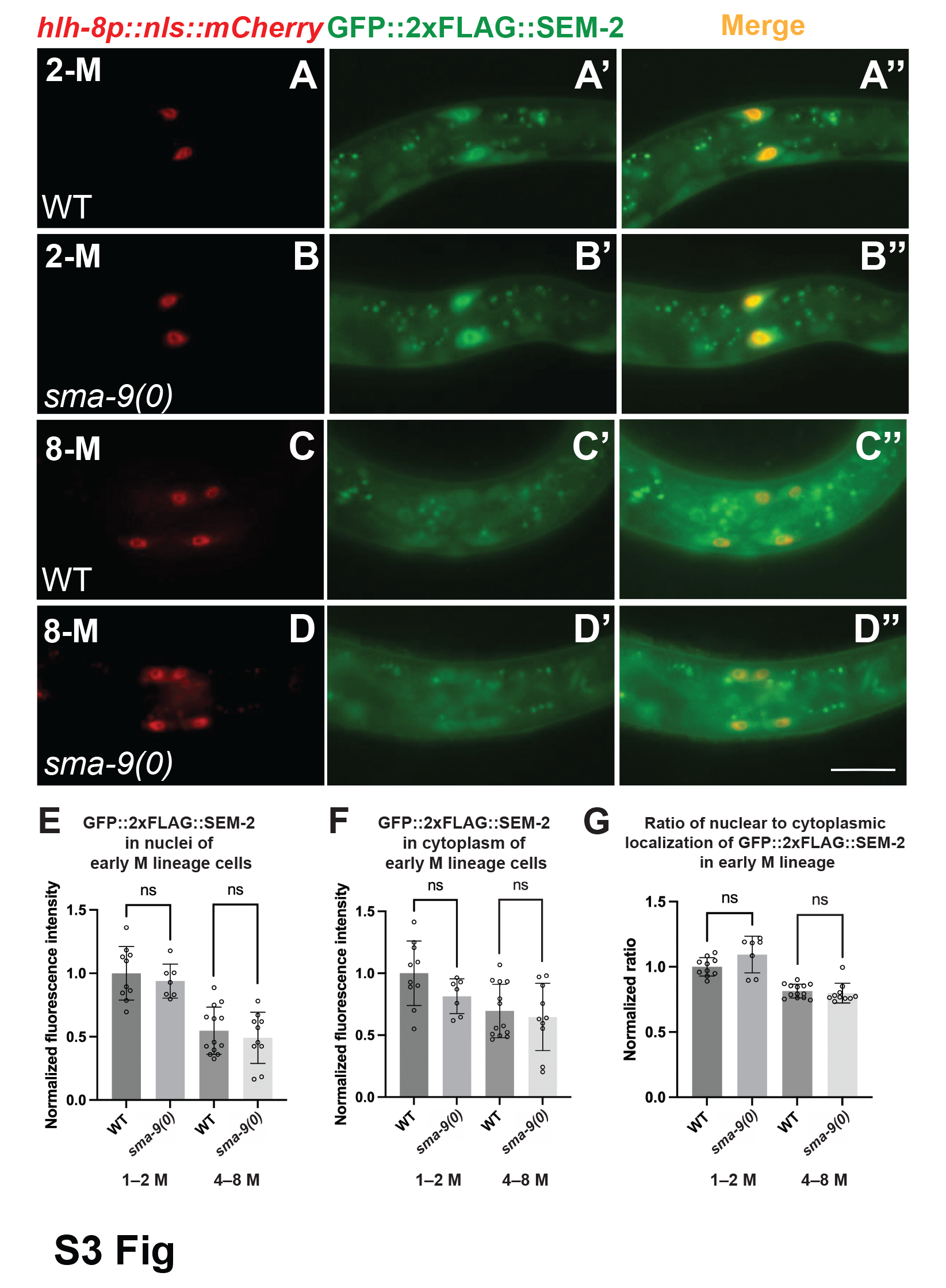

Supplement: S3 Fig — A–D”) Fluorescence images showing GFP::2xFLAG::SEM-2 (A’–D’) in M lineage cells labelled by the hlh-8p::nls::mCherry reporter (A–D) at the 2-M stage (A–B”) and 8-M stage (C–D”) of M lineage development in WT (A–A”, C–C”) and sma-9(0) (B–B”, D–D”) hermaphrodites. (A”–D”) are the corresponding merged images. Only the left side of an animal is shown in this figure, while the other side is out of the focal plane. Scale bar represents 20 μm. E–G) Quantification of GFP::2xFLAG::SEM-2 in the nuclei (E), the cytoplasm (F), and the ratio of nuclear to cytoplasmic signal (G) in the early M lineage of WT and sma-9(0) mutants. For panel G, the ratios were calculated by dividing the nuclear GFP intensity by the cytoplasmic GFP intensity. Each dot represents a cell scored. Data are normalized to WT at the 1–2 M stage. Statistical significance was calculated by performing unpaired two-tailed Student’s t-tests. ns, not significant. (TIF) [file pgen.1011361.s006.tif]

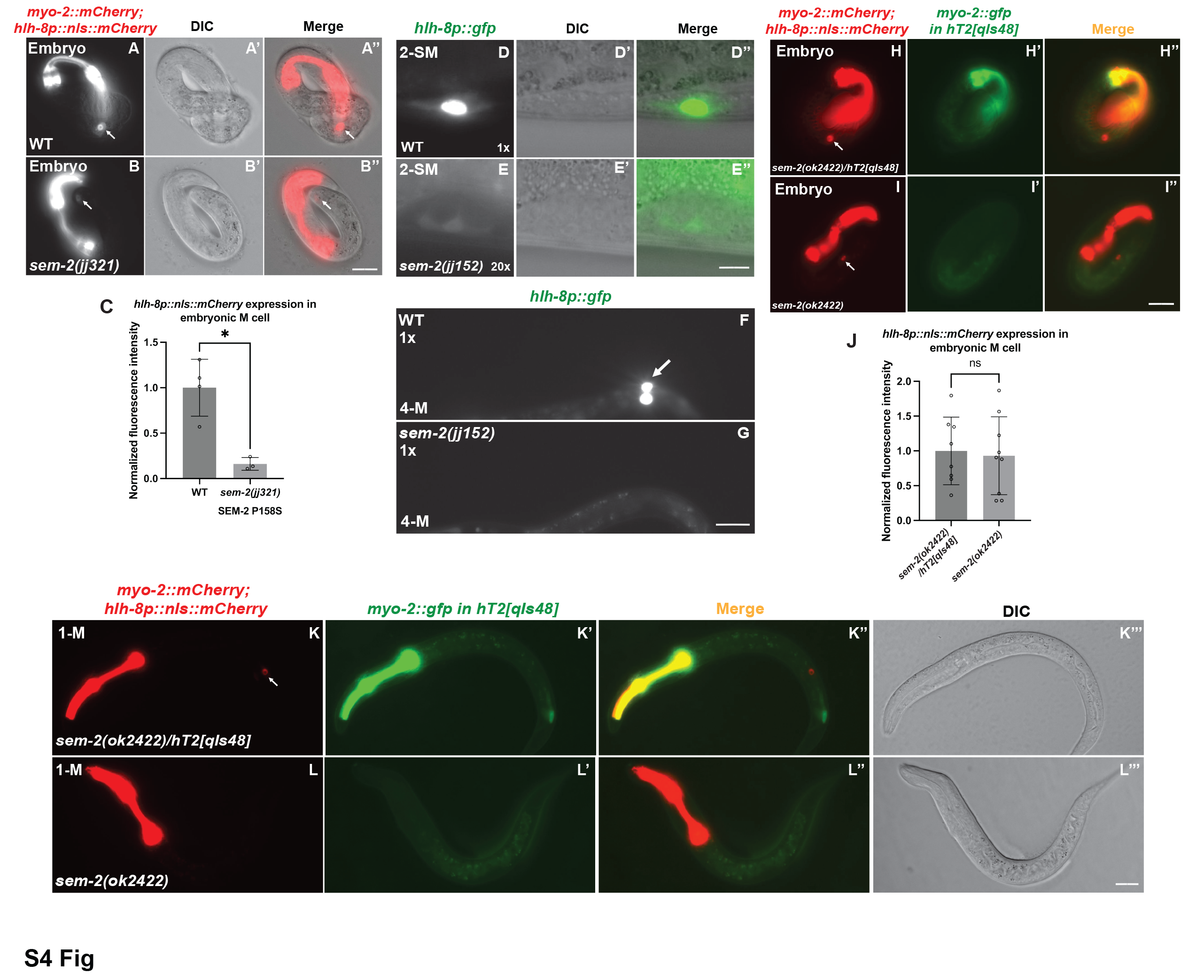

Supplement: S4 Fig — A–B”) Fluorescence images (A–B) of wild-type (A–A”) and sem-2(jj321[P158S]) (B–B”) embryos showing the expression of hlh-8p::nls::mCherry in the M mesoblast cell (arrows). A’–B’ and A”–B” are corresponding DIC and merged images, respectively. Transgenic animals expressing hlh-8p::nls::mCherry were generated with a co-injection marker myo-2p::mCherry represented by the red pharyngeal signal. Scale bar represents 15 μm. C) Quantification of hlh-8p::nls::mCherry expression in the M mesoblast cell of WT and sem-2(jj321[P158S]) embryos. Each dot represents an embryo scored. Data are normalized to WT. Statistical significance was calculated by performing unpaired two-tailed Student’s t-tests. * P<0.05. D–E) Fluorescence images of wild-type (D–D”) and sem-2(jj152[P158S]) (E–E”) mutant L3 animals showing expression of the hlh-8p::gfp transgene in SMs. D’–E’ and D”–E” are corresponding DIC and merged images, respectively. Exposure for panel E is 20x times higher than panel D (1x). Scale bar represents 10 μm. F–G) Fluorescence images of wild-type (F) and sem-2(jj152[P158S]) (G) mutant L1 animals at the 4-M stage showing expression of the hlh-8p::gfp transgene at the same exposure (1x). Scale bar represents 20 μm. H–I”) Fluorescence images (H–I) of a heterozygous sem-2 null (sem-2(ok2422)/hT2[qIs48]) (H–H”) and a sem-2 null (sem-2(ok2422)) (I–I”) embryo showing expression of hlh-8p::nls::mCherry. myo-2p::gfp images from the hT2[qIs48] balancer chromosome are shown in H’–I’ and merged images are shown in H”–I”. Scale bar represents 15 μm. J) Quantification of hlh-8p::nls::mCherry expression in the embryonic M mesoblast cell of sem-2(ok2422)/hT2[qIs48] and sem-2(ok2422) animals. Each dot represents an embryo scored. Data are normalized to sem-2(ok2422)/hT2[qIs48]. Statistical significance was calculated by performing unpaired two-tailed Student’s t-tests. ns, not significant. K–L”’) Fluorescence images (K–L) of a heterozygous sem-2 null (sem-2(ok2422)/hT2[qIs48]) (K–K”’) and a s [file pgen.1011361.s007.tif]

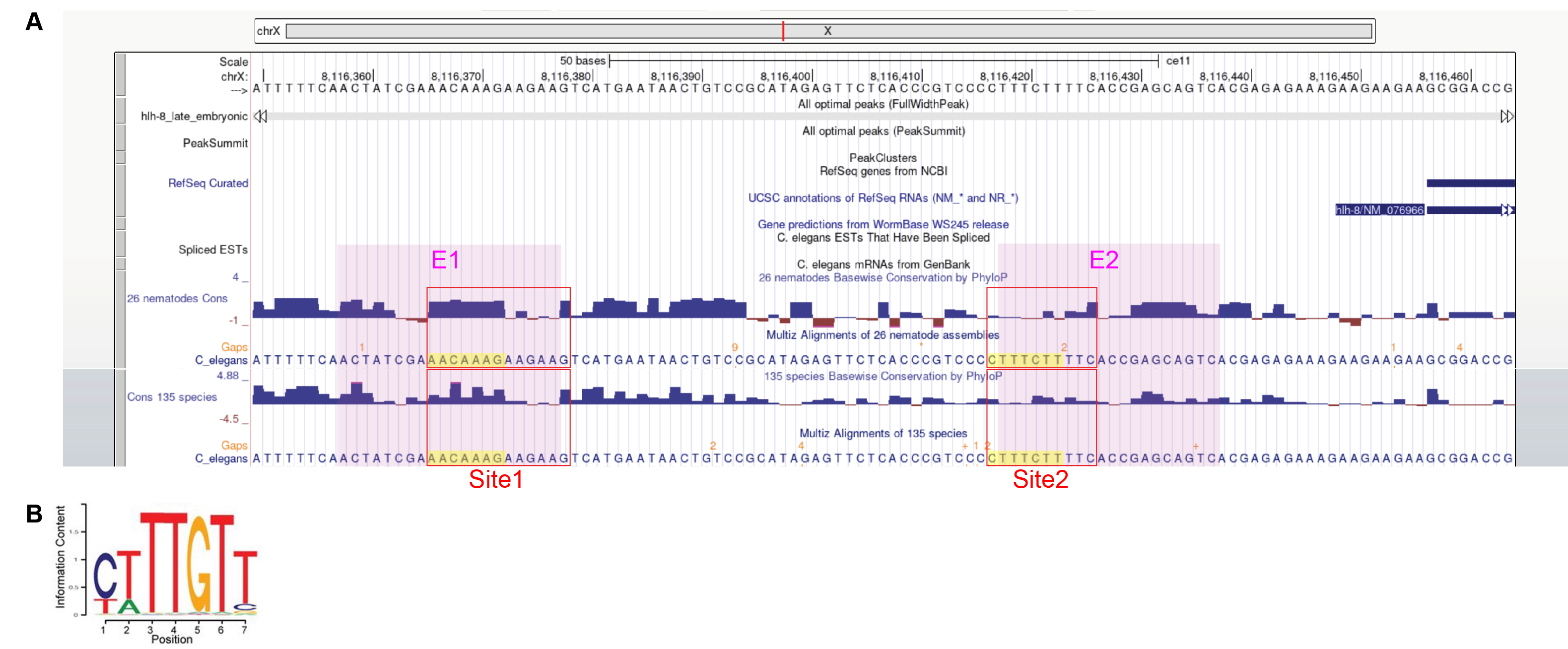

Supplement: S5 Fig — A) Screenshot of the University of California, Santa Cruz (UCSC) genome browser showing the sequence conservation of E1 and E2 in the hlh-8 promoter among 26 nematode species and a broader 135 species (112 nematodes, 22 flatworms, and Ciona intestinalis). The magenta color highlights E1 and E2. Red boxes show Site1 and Site2. The yellow color highlights the putative SEM-2/SoxC-binding sites in E1/Site1 and E2/Site2. B) The position weight matrix of the Sox4/SoxC primary motif as determined by the protein-binding microarray (PBM) method [33]. (TIF) [file pgen.1011361.s008.tif]
