# Supplementary material for: SEM-2/SoxC regulates multiple aspects of C. elegans postembryonic mesoderm development
Source: PLoS Genet. 2025 Jan 21;21(1):e1011361. doi: 10.1371/journal.pgen.1011361 (PMC11785321; doi:10.1371/journal.pgen.1011361)
Supplement: Attachment — Submitted filename: ReviewercommentsBaccasetalPLoS.pdf [file pgen.1011361.s010.pdf]

PLOS Genetics (PGENETICS-D-24-00741) - [EMID:d862d14eaf306f5d]
Baccas et al.
Title: SEM-2/SoxC regulates multiple aspects of *C. elegans* postembryonic mesoderm development

Reviewer comments:

Overview:
This manuscript reports the identification and characterization of a partial loss-of-function allele of the *C. elegans* SoxC transcription factor SEM-2 that was isolated in a screen for suppressors of a *sma-9* mutant that lacks the development of the coelomocytes in the post-embryonic development of the M lineage. The study has led to a new understanding of the relationship between SEM-2, SMA-9 and LET-381 (a FoxF/C forkhead transcription factor) and of the regulation of another transcription factor in the M lineage, HLH-8. The work has mechanistic implications regarding the transcriptional regulation of cell fate during development and potentially in the pathogenesis of disease since these factors are homologs of proteins that are defective in human developmental disorders.

General comments:
This manuscript is well-written and the experimental evidence is convincing. The comments outlines below are fairly minor except for the *hlh-8* promoter experiment that either needs the approach justified or explained further.

Specific concerns:

1. It would be helpful if Fig 1B included some additional information that depicted the relationships between the various proteins described in the introduction and the CC/SM fates that would then be built upon in the Fig 10 model.

2. The complementation test described in lines 139-141 should define qIs48—is this a GFP reporter (not indicated in the strain table either)? jjIs3900 should be described or explained. And, the isolation of red, non-green animals should be explained as well.

3. In the Plasmids section of the methods, the purpose of the plasmids should be stated before explaining how the plasmid was made.

4. In discussing the screen and identification of *jj152* (lines 217-223), it is confusing how a single allele can be a complementation group. By definition, a group should contain multiple alleles. Also, it should be stated how the *jj152* phenotypes compare with the original *sma-9* mutants.

5. In the results section, it doesn't make sense how a mutation that is predicted to not touch DNA would affect the DNA binding affinity. Consider rewording or proposing multiple possibilities.

6. In the experiments where the SoxC binding sites are being investigated in the *hlh-8* promoter there are several points that need clarifying in both the figures and the text. It is not clear why deletion assays were performed if two putative sites were initially identified. Why weren't those sites directly mutated once they were noted in the sequence analysis? Was the point of the deletion studies to see if there were any other sites in the region? Or to see whether the regions surrounding where the sites were located were necessary for *hlh-8* expression? This section of the results needs revising with more explanation. Further, it is not clear in the text or in Fig 10, what kind of mutations are being made to site 1 and site 2. Are they deletions? Are they scrambled sites (which are often better since this would maintain the spacing in the promoter)? Fig 10A should be modified to show the mutations or minimally the nature of the mutations should be stated in the legend. And, why were two site 1 mutant strains made and not a site 2 mutant strain made? Finally, in Fig 10, why are the merged panels not yellow (or at least orange) in B" and E"-G" when there appears to be signal in both of the single channels?

7. I have several other figure labeling suggestions. In Fig 3 G/H there are bright red cells in addition to the SMs, it would be helpful to have arrows marking the SMs. The legend in Figure 4 is confusing and redundant. Instead of labeling the SMs and SM-like cells white in the upper panels and yellow in the lower panels and describing them twice in the legend, just be consistent with labeling all the panels and define those labels one time in the legend.

Minor concerns and edits (with line numbers indicated):

99 and 608: "Coffin-<u>S</u>iris syndrome (CSS)"

125: "Fiji" should either be explained or have a website indicated

128: software used for statistical analysis should be indicated

152 change "give" to "produce"

153 and throughout manuscript: wild type should be two words and have a dash if modifying a noun such as wild-type animals

164: the plasmids are listed in Supp Table 3 and not 2 (fix the table numbers in lines 194 and 195 as well) or swap the tables since the text doesn't match the actual supplementary table numbers.

166: define IDT

196: pRF4 not PRF4

229: indicate the specific transgene (*jjIs1647*?)

230: Tian et al. 2011 stated here and in the discussion should be numbered like the other references.

266: say "This prediction is consistent with our genetic…"

314: after *sma-9 (0)* animals add (Fig 4E-H''')

406: "Similarly, in 50%..."

419: HLH-8 (8 not in italics) and Fig 7 A-M fix since there is no M in the figure

421: "in the vms (Fig 7 B, B'). Further, 95% *sem-2*…"

423: the text states that the mutants were variable but the Fig 7G shows a large standard deviation for WT, too, so needs revising

435: "At the late L4…"

562: *arg-1::gfp* should be in italics

588: P158S should not be in italics

814: "…Egl phenotype with a uterus filled with late-stage embryos"

820/822: "A) [Top] Schematic of the …" and "…by an asterisk. [Bottom] Sequence alignment."

882: "…mutant animal (L-M"),…"

897: "images"

926: WT should not be listed as a *sem-2* mutant in A,C,E

940: "C"-H" are merged images"

954-955: fix the excess italics

Multiple figure legends have "fluorescence" misspelled.

In Supp Table 2, last row—pMDB37 "Repair template for generating.." should not be in italics
