# Supplementary material for: SEM-2/SoxC regulates multiple aspects of C. elegans postembryonic mesoderm development
Source: PLoS Genet. 2025 Jan 21;21(1):e1011361. doi: 10.1371/journal.pgen.1011361 (PMC11785321; doi:10.1371/journal.pgen.1011361)
Supplement: Attachment — Submitted filename: Baccas et al-response to reviewers-10-5-24.pdf [file pgen.1011361.s011.pdf]

Cornell University
Molecular Biology & Genetics
439/441 Biotechnology Building
Ithaca, NY 14853, USA
Website: www.mbg.cornell.edu

Dr. Jun (Kelly) Liu
Professor
Phone: (607) 254-8942 (Office)/254-1237 (Lab)
Fax: (607) 255-6249/255-2428
E-mail: JL53@CORNELL.EDU

October 3, 2024

Professors Grant and Bosco
PLoS Genetics Editorial Office

Dear Dr. Grant and Dr. Bosco,

Enclosed please find our revised manuscript **PGENETICS-D-24-00741** entitled "**SEM-2/SoxC regulates multiple aspects of C. elegans postembryonic mesoderm development**". We very much appreciate the careful reviews from the three reviewers on our manuscript. We have revised our manuscript based on the reviewers' comments and suggestions (detailed below).

In particular, we have taken Reviewer 2's suggestions by quantifying the fluorescence intensities of almost all data presented in this manuscript, and performing statistical analysis on the measurements. We have added new figures or updated previous figures to reflect these additions. We have also made changes throughout the manuscript to address the points of all three reviewers. We have included a version of the manuscript with tracked changes to illustrate the revisions that we have made. We hope that you will find the revised manuscript appropriate for acceptance and publication.

We thank you for your time and consideration.

Sincerely,

Jun (Kelly) Liu
Professor
Molecular Biology and Genetics
Cornell University

**Reviewer #1:**
This paper describes new roles for the C. elegans SoxC protein SEM-2 during development of a postembryonic mesodermal lineage. The basis of the work is a new allele of sem-2, which is a missense mutation in the DNA binding domain that results in a partial loss of function (by genetic criteria). The authors use this mutation to further explore the function of sem-2 in M lineage specification, adding to their already significant understanding of the gene regulatory networks governing M development. They find that SEM-2 is required for the proliferation and diversification of the SM lineage, acts antagonistically with LET-381/FoxF, and (likely directly) regulates hlh-8/Twist.

This work advances our understanding of the gene regulatory mechanisms governing development of the M mesoblast, which is a postembryonic multipotent progenitor cell. This gene regulatory network may be conserved in metazoans, as homologous genes have been implicated in craniofacial development in humans. The paper is well written. The results are strong and clearly support the conclusions.

Specific points:
1. Line 64. The M mesoblast is described as pluripotent. Pluripotency is the ability to develop into all three germ layers. M is more accurately described as multipotent.

*We have changed it to “multipotent”. Thank you for pointing this out.*

2. Lines 356-359. It wasn’t clear to me why both jj321 and jj152 were examined, as both mutations result in the same amino acid change and all evidence suggests they have the same phenotype.

*The reviewer is correct, both jj321 and jj152 have the same amino acid change in sem-2. However, jj321 was generated via CRISPR, while jj152 was isolated from our sma-9 suppressor screen. For rigor and reproducibility and to rule out any background mutation(s) that may affect hlh-8p::nls::mCherry reporter expression, we examined both alleles.*

3. Lines 380-383. This sentence should be revised: “We reasoned that the more robust expression of sem-2 in sem-2(ok2422) null embryos born from [heterozygous] parents is likely due to the maternal contribution of SEM-2 by [heterozygous] parents”. I believe the authors offer an explanation as to why the phenotype of ok2422 is less severe than that of jj152. This needs to be made clearer.

*We have revised the manuscript regarding ok2422. The revised version reads: “One possible reason why there is more robust expression of hlh-8p::nls::mCherry in sem-2(ok2422) null embryos than in sem-2(jj321[P158S]) embryos is the maternal contribution of SEM-2 in the sem-2(ok2422) null embryos by sem-2(ok2422)/hT2[qIs48] parents (S4A–C, H–J Fig). Further, these results suggest that SEM-2 is required for maintaining stable hlh-8 expression in the M lineage.”*

4. Figure 5G-M. It appears that there is more background for the hlh-8::mCherry images in the sem-2 mutant background (H, L’, M’). This may be due to a longer exposure that is required because hlh-8 expression is reduced in the mutants. Exposure differences should be indicated in the figure legend.

*We have updated the legend for Figure 5 to include a sentence indicating that panels 5H, L’, M have more background due to longer exposure times.*

5. Lines 485-487. An alternative possibility is that hlh-8 does not depend on SEM-2 at later stages in the M lineage.

*We have added the following sentence. Alternatively, hlh-8 expression in the later stages of M lineage development does not depend on SEM-2.*

**Reviewer #2:**
This manuscript utilizes a novel allele of the SoxC transcription factor, sem-2, isolated in a forward genetic screen. They use a combination of CRISPR genome engineering, traditional transgene reporter analysis and imaging to characterize the phenotype of their sem-2 allele, in the context of the M lineage and the gene regulatory network controlling SM fate, proliferation and differentiation. Their results extend earlier work by their group and others in our understanding of the transcriptional network that underlies the hermaphrodite reproductive musculature.

I have several suggestions where the manuscript could be significantly improved by quantifying images rather than just reporting general trends. The authors did the appropriate quantification for some of their data (Figures 5 & 7), but this quantification is missing from the rest of the manuscript.

Major comments:

- Lines 262-265 – It would be necessary to quantify a series of images acquired using the same settings before concluding that the expression levels are the same between the wild-type and mutant GFP-tagged alleles.

*We have added a graph in the revised Figure 2E showing the quantification of GFP::2xFLAG::SEM-2 in the hypodermal cells in WT and SEM-2 P158S, indicating that the expression levels are the same between WT and SEM-2 P158S mutants.*

From the micrographs shown in Figure 3 of the endogenous expression and localization of the GFP-tagged SEM-2 allele, it appears that SEM-2 localizes to both the cytosol and nucleus. It might be important to quantify this over developmental time if possible – esp. to see if there are any differences in this localization pattern between the P158S allele. By quantifying this data, the author's could make a stronger statement, and then the shading in Figure 3L could be a quantitative read-out of the data.

*We have quantified the GFP::2xFLAG::SEM-2 signal both inside the nucleus and the cytoplasm at every stage of M lineage development in WT animals. The quantification is shown in the new Supplementary Figure 1. We have updated Figure 3L to reflect the change of GFP::2xFLAG::SEM-2 signal intensity during M lineage development. We have also updated the manuscript with the following: "At all stages of M lineage development when GFP::2xFLAG::SEM-2 is detectable, there is GFP signal in both the nucleus and the cytoplasm. The intensity of both nuclear and cytoplasmic GFP signals exhibits a gradual decrease during the two proliferative phases of M lineage development (in the early M lineage and in the SM lineage) (S1 Fig). The expression and localization pattern of GFP::2xFLAG::SEM-2 in the M lineage is quantified and is summarized in Fig 3L and S1 Fig."*

*We also quantified and compared the nuclear and cytoplasmic signals as well as the nuclear to cytoplasmic ratio of GFP::2xFLAG::SEM-2 in the M cell and the SMs between WT and SEM-2 P158S*

*animals. We have added the information in Supplemental Figure 2 (S2 Fig), and have updated the manuscript with the results of the quantification: "To determine if the P158S mutation affects the expression and/or localization of SEM-2 specifically in the M lineage, we compared the expression and localization pattern of wild-type GFP::2xFLAG::SEM-2 with mutant GFP::2xFLAG::SEM-2(P158S) (S2 Fig). At the 1-M stage, we detected a slight increase in GFP intensity as well as nuclear to cytoplasmic ratio in SEM-2 P158S mutants compared to wild-type animals (S2C,E Fig). However, there is no difference in SEM-2 level or localization in the SMs between wild-type and SEM-2 P158S mutant animals (S2F–L Fig)."*

Lines 292-296: In comparing the sem-2::GFP expression in wild-type and sma-9(cc604) backgrounds, it is difficult to assess from a single image – I would recommend quantifying a set of images before making any conclusions about expression levels between different backgrounds.

*We performed the quantification of GFP::2xFLAG::SEM-2 intensity at different stages of early M lineage development and found no difference between WT and sma-9(cc604) animals. We have updated Supplemental Figure 3 with the results of the quantification (S3E–G Fig).*

Figure S2 – I would recommend taking the same approach as you took in Figure 4 for quantifying your data. I would also recommend showing the equivalent expression comparison in panel C/D and then a label showing 20x brighter rather than just putting that information in the figure legend, as it wasn't immediately obvious to me.

*We have added images that were taken at the same exposure. We have also performed quantification of the fluorescence intensity between WT and mutant animals. The updated Supplementary Figure 4 (S4 Fig) reflects all the updates.*

When comparing expression between strains, I would suggest including the quantification rather than just the number of animals examined – for example, in line 357 it states that 100% of mutants displayed reduced expression (100%, N=234).

*In addition to the number of animals scored, the level of fluorescence intensity was also measured and quantified. We have added the information in the updated Figure 5 (Fig 5C and 5F).*

Instead I would quantify the difference and report it, rather than just the amount of a population that is in the state you are comparing too. This would help with the data and accompanying results in Figure 6 (E-G'') – I would include quantification of this data, and then report that in the text rather than the % of the population that is indicative of the state (expressing less?

*The fluorescence intensity of jj422[hlh-8p::gfp] in WT and SEM-2 P158S animals has been measured and quantified. The information is included in the updated Figure 6 (Fig 6I).*

For reporting the proliferation defect in the SM lineage in the sem-2 allele (Figure 8 and accompanying text), I think a summary figure or accompanying plot showing the number of SMs per stage would be appropriate – you could stage animals in sem-2 mutants by VPC divisions.

*We have monitored the M lineage division patterns in WT and SEM-2 P158S mutants, and found that the mutants exhibit variable patterns of reduced SM proliferation. We have updated the manuscript*

*and Figure 8 (Fig 8G), which summarizes the different types of SM division patterns observed in sem-2(jj321[P158S]) mutants.*

Discussion –

Line 550-551 – Rather than state that sem-2 might interact with cell cycle regulators interacting with proteins mentioned in a specific review, I would be specific here – possibly through positive regulation of G1/S-phase cyclins and CDKs (Cyclin D/CDK4, Cyclin E/CDK2) or possibly negative regulation of CDK inhibitors like CKIs (CKI-1/CKI-2)?

*We appreciate the reviewer's suggestion. We have added this to the revised manuscript.*

Minor comments:

Scale bars are missing in the micrographs

*We have added the scale bars in all the micrographs.*

For most of the figures, I would recommend showing single color images in grey scale as the human eye can see more details in gray scale than in color. For the overlays, it would be good to avoid red/green combinations as many people are color blind – you could use magenta for red, or any other color-blind friendly look-up-table (LUT) for your overlays. The data was shown this way in some of the figures, but I would recommend doing this for all single channel images, reserving false colors for overlays.

*We thank the reviewer for this suggestion. We will definitely take the advice moving forward, using gray scale for single channel images and color-blind friendly combinations for overlays in our future manuscripts. For this paper, we think that the signals in the single channel images that have color are bright enough to show the details, while those images that have fainter signals are already shown in gray scale.*

**Reviewer #3:**
Overview:
This manuscript reports the identification and characterization of a partial loss-of-function allele of the *C. elegans* SoxC transcription factor SEM-2 that was isolated in a screen for suppressors of a *sma-9* mutant that lacks the development of the coelomocytes in the post-embryonic development of the M lineage. The study has led to a new understanding of the relationship between SEM-2, SMA-9 and LET-381 (a FoxF/C forkhead transcription factor) and of the regulation of another transcription factor in the M lineage, HLH-8. The work has mechanistic implications regarding the transcriptional regulation of cell fate during development and potentially in the pathogenesis of disease since these factors are homologs of proteins that are defective in human developmental disorders.

General comments:
This manuscript is well-written and the experimental evidence is convincing. The comments outlines below are fairly minor except for the *hlh-8* promoter experiment that either needs the approach justified or explained further.

Specific concerns:

1. It would be helpful if Fig 1B included some additional information that depicted the relationships between the various proteins described in the introduction and the CC/SM fates that would then be built upon in the Fig 10 model.

*Thanks for the suggestion! We have modified Figure 1 to include the following: 1) marking cells that express SMA-9, LIN-12, LET-381, CEH-34, and SEM-2 in the early M lineage leading up to the birth of the CCs and SMs. 2) adding a panel showing how these afore mentioned factors function to regulate CC and SM fate specification based on our previously published work. Figure 1 legend has also been accordingly updated in the revised manuscript.*

2. The complementation test described in lines 139-141 should define qIs48—is this a GFP reporter (not indicated in the strain table either)? jjIs3900 should be described or explained. And, the isolation of red, non-green animals should be explained as well.

*We have added an explanation for the complementation test and the detailed information about qIs48 and jjIs3900 in Materials and Methods.*

3. In the Plasmids section of the methods, the purpose of the plasmids should be stated before explaining how the plasmid was made.

*We have modified the Plasmids section of the methods to describe the purpose of the plasmids in the revised manuscript.*

4. In discussing the screen and identification of *jj152* (lines 217-223), it is confusing how a single allele can be a complementation group. By definition, a group should contain multiple alleles. Also, it should be stated how the *jj152* phenotypes compare with the original *sma-9* mutants.

*We have modified the sentence in the revised manuscript when discussing the identification of jj152. We also added a sentence to explain that jj152 mutants do not have a small body size, and have the normal number of CCs.*

5. In the results section, it doesn't make sense how a mutation that is predicted to not touch DNA would affect the DNA binding affinity. Consider rewording or proposing multiple possibilities.

*It has been well established that for transcription factors, mutations of residues in regions that do not directly touch DNA can change the conformation of the transcription factor, thus affecting its DNA binding affinity. However, it is also possible that the P158S mutation could affect the binding of SEM-2 to other factors. We have modified the manuscript to reflect these other possibilities as well.*

6. In the experiments where the SoxC binding sites are being investigated in the *hlh-8* promoter there are several points that need clarifying in both the figures and the text. It is not clear why deletion assays were performed if two putative sites were initially identified. Why weren't those sites directly mutated once they were noted in the sequence analysis? Was the point of the deletion studies to see if there were any other sites in the region? Or to see whether the regions surrounding where the sites were located were necessary for *hlh-8* expression? This section of the results needs revising with

more explanation. Further, it is not clear in the text or in Fig 10, what kind of mutations are being made to site 1 and site 2. Are they deletions? Are they scrambled sites (which are often better since this would maintain the spacing in the promoter)? Fig 10A should be modified to show the mutations or minimally the nature of the mutations should be stated in the legend. And, why were two site 1 mutant strains made and not a site 2 mutant strain made? Finally, in Fig 10, why are the merged panels not yellow (or at least orange) in B" and E"-G" when there appears to be signal in both of the single channels?

*The reviewer is correct that we performed the deletion analysis to not only test whether SEM-2 directly regulates hlh-8 expression but also to identify all sequences that are required for hlh-8 expression in the M lineage. We have revised this part of the manuscript to reflect this intention.*

*We have also explained more clearly in the revised manuscript and in modified Figure 10A the nature of the site 1 and site 2 mutations that we made--they are base pair substitutions not deletions, to maintain the correct spacing in the promoter--and why we made mutations in Site1 alone and Site1 and Site2 together--because Site1 has an exact match to the SoxC binding consensus.*

*We have also updated Figure 10 to reflect the correct merged color of the red and green fluorescence signals. We apologize that the original merged images had one color overpowering the other.*

7. I have several other figure labeling suggestions. In Fig 3 G/H there are bright red cells in addition to the SMs, it would be helpful to have arrows marking the SMs. The legend in Figure 4 is confusing and redundant. Instead of labeling the SMs and SM-like cells white in the upper panels and yellow in the lower panels and describing them twice in the legend, just be consistent with labeling all the panels and define those labels one time in the legend.

*Thanks for the suggestion. We have made the recommended changes in the modified Figure 4 and Figure 4 legend in the revised manuscript.*

Minor concerns and edits (with line numbers indicated):

99 and 608: "Coffin-Siris syndrome (CSS)"

*Fixed*

125: "Fiji" should either be explained or have a website indicated

*We have added a reference about Fiji.*

128: software used for statistical analysis should be indicated

*We have added the name of the software used for statistical analyses in Materials and Methods.*

152 change "give" to "produce"

*Fixed*

153 and throughout manuscript: wild type should be two words and have a dash if modifying a noun such as wild-type animals

*Fixed*

164: the plasmids are listed in Supp Table 3 and not 2 (fix the table numbers in lines 194 and 195 as well) or swap the tables since the text doesn't match the actual supplementary table numbers.

*We have fixed this and the revised version has the correct tables referenced in the text.*

166: define IDT

*Fixed*

196: pRF4 not PRF4

*Fixed*

229: indicate the specific transgene (*jjIs1647*?)

*We have added the transgene information in the revised manuscript.*

230: Tian et al. 2011 stated here and in the discussion should be numbered like the other references.

*Fixed*

266: say "This prediction is consistent with our genetic…"

*Fixed*

314: after *sma-9 (0)* animals add (Fig 4E-H''')

*Fixed*

406: "Similarly, in 50%…"

*Fixed*

419: HLH-8 (8 not in italics) and Fig 7 A-M fix since there is no M in the figure

*Fixed*

421: "in the vms (Fig 7 B, B'). Further, 95% *sem-2*…"

*Fixed*

423: the text states that the mutants were variable but the Fig 7G shows a large standard deviation for WT, too, so needs revising

*We have revised the manuscript to reflect the variability of egl-15p::rfp fluorescence intensity in both WT and sem-2(jj152) mutants, and the slight reduction egl-15p::rfp fluorescence intensity as well as the significant reduction of the number of egl-15p::rfp-expressing cells in sem-2(jj152) mutants*.

435: “At the late L4…”

*Fixed*

562: *arg-1::gfp* should be in italics

*Fixed*

588: P158S should not be in italics

*Fixed*

814: “…Egl phenotype with a uterus filled with late-stage embryos”

*Fixed*

820/822: “A) [Top] Schematic of the …” and “…by an asterisk. [Bottom] Sequence alignment.”

*Fixed*

882: “…mutant animal (L-M”),…”

*Fixed*

897: “images”

*Fixed*

926: WT should not be listed as a *sem-2* mutant in A,C,E

*Fixed*

940: “C”-H” are merged images”

*Fixed*

954-955: fix the excess italics

*Fixed*

Multiple figure legends have “fluorescence” misspelled.

*Fixed*

In Supp Table 2, last row—pMDB37 “Repair template for generating..” should not be in italics

*Fixed*
